# Acetylation by the Transcriptional Coactivator Gcn5 Plays a Novel Role in Co-Transcriptional Spliceosome Assembly

**DOI:** 10.1371/journal.pgen.1000682

**Published:** 2009-10-16

**Authors:** Felizza Q. Gunderson, Tracy L. Johnson

**Affiliations:** Department of Biology, Molecular Biology Section, University of California San Diego, La Jolla, California, United States of America; University of California San Francisco, United States of America

## Abstract

In the last several years, a number of studies have shown that spliceosome assembly and splicing catalysis can occur co-transcriptionally. However, it has been unclear which specific transcription factors play key roles in coupling splicing to transcription and the mechanisms through which they act. Here we report the discovery that Gcn5, which encodes the histone acetyltransferase (HAT) activity of the SAGA complex, has genetic interactions with the genes encoding the heterodimeric U2 snRNP proteins Msl1 and Lea1. These interactions are dependent upon the HAT activity of Gcn5, suggesting a functional relationship between Gcn5 HAT activity and Msl1/Lea1 function. To understand the relationship between Gcn5 and Msl1/Lea1, we carried out an analysis of Gcn5's role in co-transcriptional recruitment of Msl1 and Lea1 to pre-mRNA and found that Gcn5 HAT activity is required for co-transcriptional recruitment of the U2 snRNP (and subsequent snRNP) components to the branchpoint, while it is not required for U1 recruitment. Although previous studies suggest that transcription elongation can alter co-transcriptional pre-mRNA splicing, we do not observe evidence of defective transcription elongation for these genes in the absence of Gcn5, while Gcn5-dependent histone acetylation is enriched in the promoter regions. Unexpectedly, we also observe Msl1 enrichment in the promoter region for wild-type cells and cells lacking Gcn5, indicating that Msl1 recruitment during active transcription can occur independently of its association at the branchpoint region. These results demonstrate a novel role for acetylation by SAGA in co-transcriptional recruitment of the U2 snRNP and recognition of the intron branchpoint.

## Author Summary

Pre-messenger RNA splicing, the removal of non-coding RNA sequences (introns) that interrupt the protein-coding sequence of genes, is required for proper gene expression. While recent studies have revealed that intron recognition begins while the RNA is actively being synthesized by RNA polymerase II, little is known about how the proteins involved in gene transcription and RNA splicing interact to coordinate the two reactions. Here we show that the protein complex SAGA, which allows RNA polymerase II to navigate the three-dimensional structure of packaged DNA by acetylating histone proteins, has an additional role in pre-messenger RNA splicing. Our genetic analysis shows that the SAGA complex has functional interactions with specific components of the splicing machinery. Furthermore, SAGA's acetylation activity, which we find to be targeted toward promoter-bound histones of intron-containing genes, is required for proper recruitment of these components to RNA during active transcription. Our work supports a model whereby SAGA–dependent acetylation facilitates recruitment of the splicing machinery to the pre–mRNA for proper co-transcriptional splicing.

## Introduction

Eukaryotic genes are interrupted by stretches of noncoding sequence (introns), which are removed from the newly-synthesized RNA by the spliceosome, a dynamic ribonucleoprotein complex made up of 5 highly structured snRNAs and over a hundred snRNA-associated proteins.

Although RNA synthesis and RNA splicing have been analyzed as biochemically separate reactions, recent studies demonstrate that these processes are spatially and temporally coordinated [1]. *In vivo*, recognition of splice sites within the pre-mRNA by the spliceosome can occur while the polymerase is actively engaged with the DNA template [2]–[6], and recent chromatin IP studies (in yeast and in mammals) suggest that this recruitment, or at least the *stable* association of snRNPs with the transcription complex, occurs in response to synthesis of specific signals in the pre-messenger RNA [7]–[10]. The regulatory implications of this coordination are suggested by studies showing that changes in transcription elongation caused by changes in the activity of specific transcription factors or the presence of transcriptional inhibitors can affect the spliceosome's recognition of splice sites [11],[12]. These studies focus on the spliceosome's use of alternative splice sites in response to transcription signals, but they raise the possibility that constitutive splicing signals are also affected by conditions or factors that modulate transcription. Despite the evidence that co-transcriptional spliceosome assembly occurs, there is much to learn about the mechanism whereby splicing factors are co-transcriptionally recruited.

Transcription of DNA is strongly influenced by its packaging. The core histone proteins, H2A, H2B, H3, and H4 form an octameric complex that DNA is wrapped around to form the nucleosome, which is further compacted into chromatin—a general repressor of transcription. However, histones undergo extensive post-translational modifications on their N-terminal tails including acetylation, ubiquitination, methylation, and phosphorylation, which alter the chromatin and, in turn, affect transcription. One of the best-characterized histone modifications is the reversible acetylation of lysine residues on the N-terminal tails of histones H2B, H3, and H4. Histone acetylation, which is a positive mark of transcription, neutralizes the charge on the basic histone proteins leading to relaxation of the protein/DNA interactions, and the acetylated histone tails can serve as binding sites for proteins that regulate transcription.

Histone acetylation is carried out by several different acetyltransferases, the best characterized of which is the protein Gcn5, a component of the multi-subunit transcription co-activating SAGA (Spt/Ada/Gcn5/Ada) complex (STAGA in mammals). Gcn5 primarily acetylates histones H3 and H2B, and these modifications are thought to loosen chromatin for specific transcription factor binding. Furthermore, association between the SAGA complex and general transcription factors, such as TBP, facilitate preinitiation complex formation [13],[14]. Gcn5 affects global acetylation of histones throughout the genome [15], but is typically found at the promoter and within coding regions and can influence elongation in addition to events at the promoter [16].

The co-transcriptional nature of pre-messenger RNA splicing raises the intriguing possibility that proteins involved in transcription and histone modification might affect splicing and its regulation. In fact, biochemical studies using mammalian cells indicate that histone-modifying enzymes that regulate histone acetylation physically interact with splicing factors. Prp4K, a U5 snRNP-associated kinase, copurifies with N-CoR, a nuclear hormone corepressor complex that mediates histone deacetylase activity and the mammalian chromatin remodeling protein Brg1 [17]. In an independent affinity purification/mass spectrometry analysis, N-CoR was also found associated with SAP130 and SF3a120, components of the U2 snRNP that stabilize U2 snRNP-branchpoint interactions [18]. Interestingly, SAP130 also copurifies with the human STAGA complex containing hGcn5 [19]. These studies suggest that mammalian complexes that regulate histone acetylation and chromatin remodeling have physical interactions with splicing factors, although the nature of these interactions remains unclear.

Based upon the spatial and temporal proximity of chromatin, chromatin-modifying enzymes (such as Gcn5), and pre-mRNA splicing complexes during gene expression, we undertook an analysis of genetic interactions between *GCN5* and genes encoding nonessential splicing factors. Here we show that deletion of the gene encoding Gcn5 (and not other yeast lysine acetyltransferases that target histones) is synthetically lethal when combined with deletion of either gene encoding the U2 snRNP proteins Lea1 and Msl1 (mammalian U2A′/U2B″). A mutation in *GCN5* that eliminates the protein's catalytic activity is sufficient to confer the synthetic lethality. Co-transcriptional recruitment of the U2 snRNP to the branchpoint and subsequent steps in spliceosome assembly are dependent on Gcn5 HAT activity. While previous studies indicate that transcription elongation can alter co-transcriptional spliceosome assembly, chromatin IP results reveal no obvious changes in elongation in the absence of Gcn5's HAT activity. Moreover, we observe a dramatic peak in Gcn5-dependent acetylation of histone H3 in the promoters of these intron-containing genes. Unexpectedly, we also find recruitment of Msl1 at the promoter region, indicating that Msl1 recruitment during active transcription can occur independently of its association at the branchpoint region. These results demonstrate a novel role for acetylation by SAGA in co-transcriptional recruitment of the U2 snRNP and recognition of the intron branchpoint.

## Results

### The genes encoding the U2 snRNP components Msl1 and Lea1 interact genetically with the gene encoding the histone acetyltransferase Gcn5

**Figure 1:**
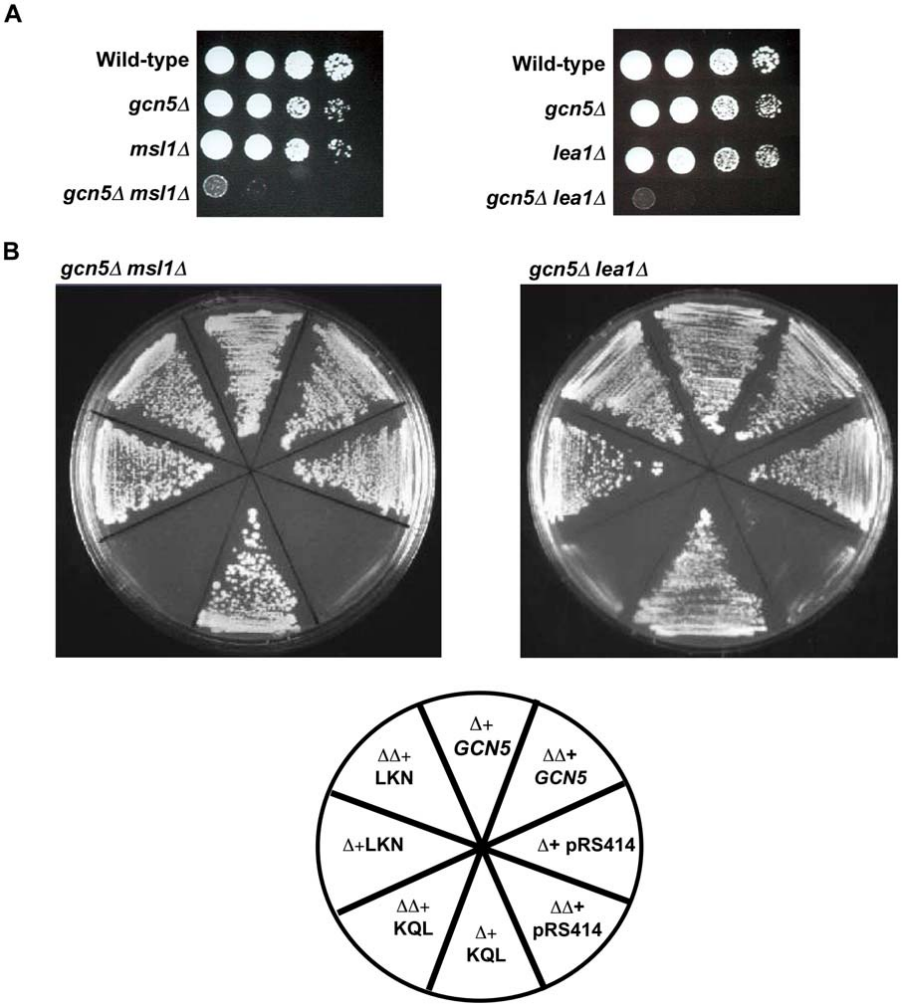
*GCN5* interacts with genes encoding the non-essential U2 snRNP proteins, *MSL1* and *LEA1*. (A) Dilution series of double mutants, *gcn5Δ msl1Δ* and *gcn5Δ lea1Δ*. Cells were grown at 30°C in YPD liquid medium until the desired O.D._600_ was obtained. Cells were spotted onto YPD plates as a ten-fold serial dilution, and the plates were incubated at 30°C for two days. (B) Viability analysis of the double mutants *gcn5Δ msl1Δ* and *gcn5Δ lea1Δ* in the presence of Gcn5 mutants. Cells were transformed with *GCN5* HAT mutants (*TRP* plasmids, pRS314) and then streaked onto 5-FOA-TRP to select for the ability to lose the wild-type copy of *GCN5* on a *URA3*-marked plasmid. Plates were incubated at 30°C for two days. Δ indicates deletion of *GCN5*; ΔΔ indicates deletion of *GCN5* and either *MSL1* or *LEA1*.

In order to characterize interactions between the non-essential histone acetyltransferase *GCN5* and genes encoding factors involved in pre-mRNA splicing, a targeted genetic screen was performed to identify synthetic lethal interactions between null alleles of non-essential splicing factors and *GCN5*. In this analysis, we uncovered genetic interactions between *GCN5* and the splicing factors *MSL1 and LEA1* (Figure 1A).

Msl1 and Lea1 are the yeast homologs of the human U2 snRNP proteins U2A′/B″ and, like their mammalian counterparts, are components of the U2 snRNP that bind to a conserved stem-loop structure in the U2 snRNA (Stem-loop IV) [20]. *In vitro*, spliceosome assembly is blocked prior to addition of the U2 snRNP in the absence of either Lea1 or Msl1, indicating a role for these proteins in U2 snRNA association with the pre-mRNA. Cells deleted of either gene also have a mild growth defect, which is observable in the strain background used here.

To determine if Gcn5's catalytic activity is required for the interactions with the genes encoding Msl1 and Lea1, we analyzed specific mutants in the HAT domain of Gcn5. A previously characterized mutation in *GCN5* which changes amino acids 126–128 (KQL) in domain I to alanines eliminates the histone acetyltransferase activity of Gcn5 [21]. The effect of this allele was tested in the double mutants, and the KQL mutant is unable to support growth of either *gcn5*Δ *msl1*Δ or *gcn5*Δ *lea1*Δ double mutant (Figure 1B). By contrast, a mutation in the same domain that changes amino acids 120–122 (LKN) to alanines and does not affect Gcn5 HAT activity [21] supports growth of the double mutants (Figure 1B). These results demonstrate that the acetyltransferase activity of Gcn5 is critical for the functional interactions with Msl1 and Lea1.

**Figure 2:**
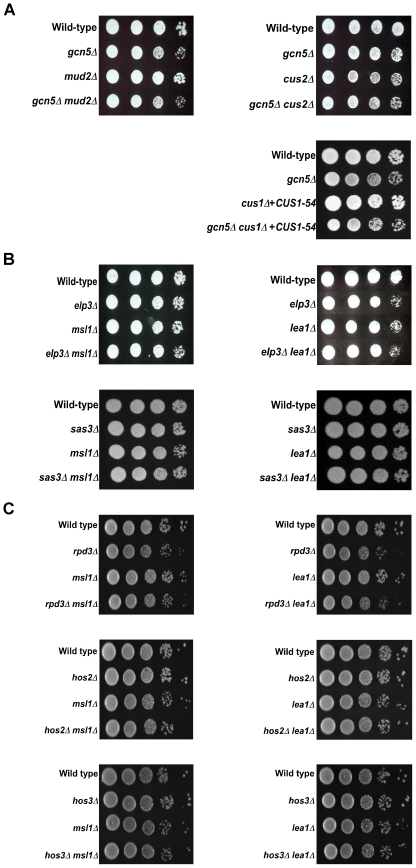
*GCN5* genetic interactions with *MSL1* and *LEA1* are specific. (A) Dilution series of double mutants *gcn5Δ mud2Δ, gcn5Δ cus2Δ and gcn5Δcus1Δ+CUS1−54*. Cells were grown at 30°C in YPD liquid medium until the desired O.D._600_ was obtained. Cells were spotted onto YPD plates as a ten-fold serial dilution. Plates were incubated at 30°C for two days. (B) Dilution series of the double mutants *elp3Δ msl1Δ*, *elp3Δ lea1Δ*, *sas3Δ msl1Δ*, *sas3Δ lea1Δ*. Cells were treated as described in (A). (C) Dilution series of the double mutants *rpd3D msl1D*, *rpd3D lea1D*, *hos2D msl1D*, *hos2D lea1D hos3D msl1D*, and *hos3D lea1D*. Cells were treated as described in (A).

We also tested other factors that have interactions with Msl1 and Lea1 and are involved in branchpoint recognition, including the commitment complex protein Mud2, and the U2 snRNP proteins Cus2 and Cus1, and found no genetic interactions between these factors and *GCN5* (Figure 2A). These results demonstrate specificity in the interaction between *GCN5* and *MSL1* or *LEA1*. While we cannot exclude the possibility that there are other essential components of the U2 snRNP that interact with *GCN5*, the effect is not general for all splicing factors acting at the prespliceosome formation step.

In addition to Gcn5, there are several other HATs that affect gene expression in yeast, including Elp3, the catalytic component of the elongator complex, and Sas3, a component of the NuA3 complex. While both histone acetyltransferases share substrates with Gcn5, and deletion of either gene is synthetically lethal when combined with deletion of *GCN5* [22], neither *ELP3* deletion nor *SAS3* deletion has a synthetic interaction with *LEA1* or *MSL1* (Figure 2B), suggesting that the interactions between *GCN5* and *MSL1* and *LEA1* are specific to the activity of Gcn5 and are not a general feature of all histone acetyltransferases.

In addition to acetyltransferases, several deacetylases have been shown to act on the same histone residues as Gcn5. The histone deacetylase Rpd3 regulates transcription and silencing, and has genetic interactions with Gcn5 [23]. Additionally, Hos2 and Hos3 are involved in gene activation and have been shown to deacetylate histones within the body of genes [16],[24]. Mutation of *HOS2* suppresses *gcn5*Δ *elp3*Δ phenotypes [22]. When deletion of *RPD3*, *HOS2*, or *HOS3* is combined with deletion of *MSL1* or *LEA1*, cells grow indistinguishably from either deletion alone (Figure 2C), suggesting that the acetylation activity of Gcn5 is functionally related to the activities of Msl1/Lea1, while the removal of acetyl groups from histones probably is not.

### *MSL1* and *LEA1* have genetic interactions with structural components of SAGA

SAGA is a 1.8 MDa, multisubunit complex comprised of five domains containing distinct sets of subunits [25]. Interactions between Msl1 and Lea1 and these other components of the complex were also analyzed and are summarized in Table 1. Ada2 and Ada3 directly interact with Gcn5, are required for Gcn5 catalytic activity, and direct Gcn5's histone acetylation activity toward nucleosomes [26]–[29]. We hypothesized that, since abrogation of the catalytic activity of *GCN5* leads to synthetic lethality in cells deleted of *MSL1* and *LEA1*, a similar synthetic growth defect would be evident in the *ada2*Δ *msl1*Δ or the *ada2*Δ *lea1*Δ mutants, and indeed, this is what is observed. Furthermore, deletion of *SPT7*, which is required for the structural integrity of the SAGA complex [25],[30],[31], is lethal when combined with deletion of either *MSL1 or LEA1*, indicating that the interactions occur within the context of a functional complex. Two components of SAGA that target the complex to the promoter, Spt3 and Spt8 [31]–[33], also have genetic interactions with Msl1 and Lea1. Spt8 is unique to the SAGA complex and is missing from the other Gcn5 containing complexes, SALSA and SILK [34], suggesting that the interactions between *GCN5* and *MSL1* and *LEA1* occur within the context of the SAGA and not the SALSA or SILK complexes. Deletion of genes encoding other components of SAGA that do not contribute to SAGA's HAT activity, such as Ubp8 or Sgf11, show no synthetic growth defects when combined with deletion of *GCN5*. Taken together, these data strongly suggest that the intact SAGA complex, with its catalytic activity targeted to nucleosomes, has a functional interaction with Msl1 and Lea1.

**Table 1:** Summary of genetic interactions between U2 snRNP factors, Msl1/Lea1, and SAGA components.

	Double Mutant	Phenotype
**SAGA Catalytic Module**	*ada2*Δ *msl1*Δ	Synthetic lethality
	*ada2*Δ *lea1*Δ	Synthetic lethality
	*ada3*Δ *msl1*Δ	Severe growth defect
	*ada3*Δ *lea1*Δ	Severe growth defect
**SAGA Structural Integrity**	*spt7*Δ *msl1*Δ	Severe growth defect
	*spt7*Δ *lea1*Δ	Severe growth defect
**TBP Recruitment**	*spt3*Δ *msl1*Δ	Severe growth defect
	*spt3*Δ *lea1*Δ	Severe growth defect
	*spt8*Δ *msl1*Δ	Severe growth defect
	*spt8*Δ *lea1*Δ	Severe growth defect
**Ubiquitination**	*ubp8*Δ *msl1*Δ	No growth defect
	*ubp8*Δ *lea1*Δ	No growth defect
	*sgf11*Δ *msl1*Δ	No growth defect
	*sgf11*Δ *lea1*Δ	No growth defect

The genotype of each spore was confirmed by PCR, as described in Materials and Methods.

The best-characterized substrates of Gcn5 are lysine residues on histones, suggesting a model in which chromatin modification has some overlapping function with pre-mRNA splicing factors. Nonetheless, we considered the possibility that the genetic interactions we observed between *GCN5* and *MSL1* and *LEA1* are due to Gcn5's catalytic activity being directed toward one of these non-histone substrates. Using an antibody that recognizes acetylated lysine residues we probed an immunoprecipitated Lea1-HA sample and an Msl1-HA sample to detect acetylation of these proteins in the presence or absence of Gcn5 and do not detect acetylation of either protein or associated U2 snRNP proteins (data not shown). While this does not rule out the possibility that Gcn5 acetylates some other splicing factor, these data do suggest that the genetic interactions between *GCN5* and *MSL1* and *LEA1* are probably not due to acetylation of the U2 snRNP proteins by Gcn5, and indicate a novel functional interaction between the transcriptional co-activator complex, SAGA, and core components of the spliceosome.

### Deletion of *GCN5* abrogates co-transcriptional recruitment of Lea1 and Msl1

Recent studies in yeast demonstrate that *in vivo* spliceosome recruitment to pre-mRNA occurs while the nascent RNA is actively engaged with the transcription complex [8]. Chromatin immunoprecipitation provides a powerful tool for detecting this co-transcriptional recruitment. The individual snRNPs can be formaldehyde crosslinked to the transcription complex or to the nascent RNA and immunoprecipitated. When the associated DNA is amplified, the signal is enriched in regions of the gene where the snRNPs would be predicted to associate, in a stepwise manner, with the corresponding pre-mRNA [8].

**Figure 3:**
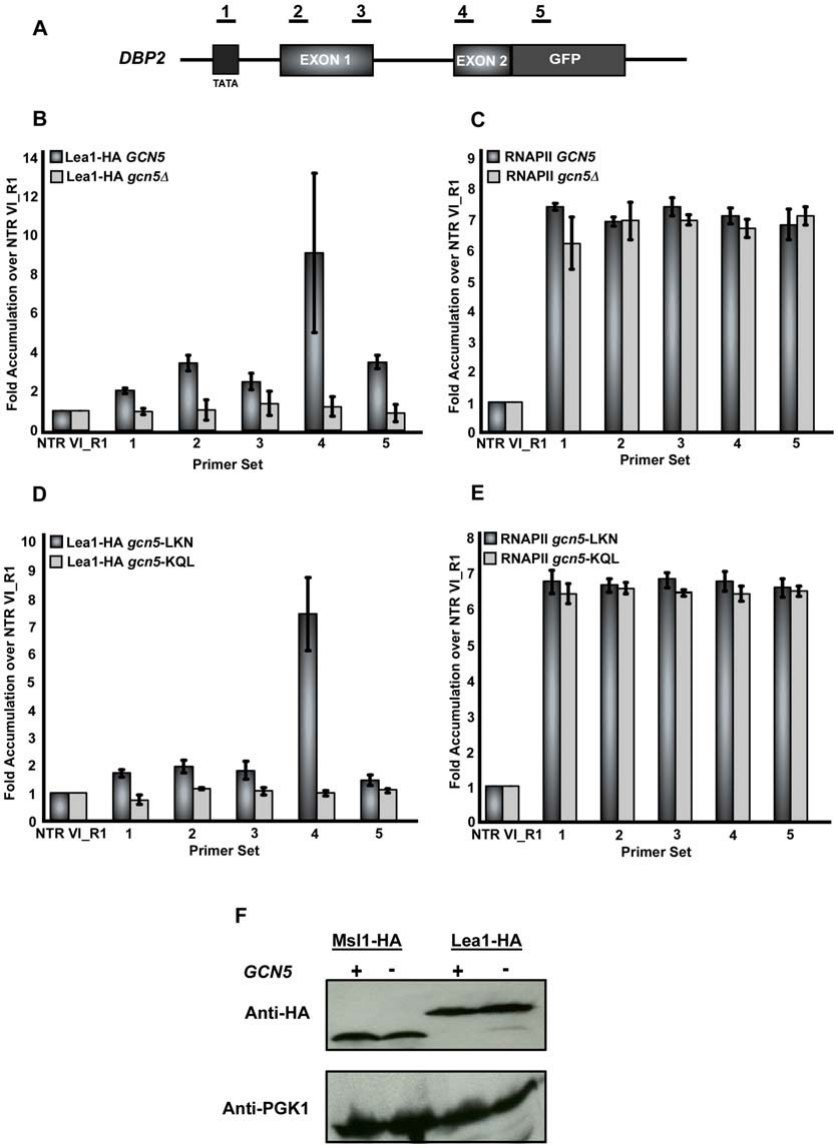
Deletion of *GCN5* affects co-transcriptional recruitment of Lea1 to *DBP2*. (A) Schematic of the intron-containing gene, *DBP2*. Underlined numbers represent amplicons generated from each primer set used in the study. (B) Graph depicting the occupancy of Lea1 at each region of *DBP2* relative to the non-transcribed region, in wild-type or *gcn5Δ*. Dark grey bars represent Lea1 with wild-type *GCN5* and light grey bars represent Lea1 levels in the *gcn5Δ* strain. (C) Bar graph depicting RNA pol II occupancy within *DBP2* relative to the non-transcribed control. Dark grey bars represent RNAP II occupancy in the *LEA1-HA* strain and light grey bars represent RNAP II occupancy in the *LEA1-HA gcn5*Δ strain. (D) Graph depicting the occupancy of Lea1 with the Gcn5 HAT mutants, LKN and KQL. Dark grey bars represent Lea1 with the Gcn5 LKN mutation, light grey bars represent Lea1 with the Gcn5 KQL mutation. (E) Bar graph depicting RNA pol II occupancy in the presence of the Gcn5 HAT mutants. Dark grey bars represent RNA pol II occupancy with the Gcn5 LKN mutation and light grey bars represent RNAP II with the Gcn5 KQL mutant. All graphs depict the average of at least three independent experiments, and error bars represent the standard deviation. (F) Protein Immunoblot of strains used for ChIP assays. Wild-type and *gcn5Δ* cultures were grown in YPD liquid medium and whole cell extracts were prepared (see Material and Methods) and probed with anti-HA 12CA5 (Roche), shown in the top panel. Extracts were also probed with anti-PGK1 (Invitrogen/Molecular Probes) as a loading control (bottom panel).

To determine if co-transcriptional recruitment of either Msl1 or Lea1 is affected by deletion of *GCN5*, we analyzed the well-characterized intron-containing gene *DBP2* with an extended exon 2 (Figure 3A). In strains in which *GCN5* is present, we detect Lea1 recruitment after synthesis of the pre-mRNA branchpoint sequence (Figure 3B), a result consistent with what has been reported by others [8]. However, when *GCN5* is deleted, there is a dramatic decrease in Lea1 association with *DBP2* (Figure 3B). RNA polymerase association along *DBP2* was also examined, and no significant difference between the levels of RNA polymerase at the 5′ and 3′ ends of *DBP2* are apparent when *GCN5* was deleted. In fact, the polymerase distribution along the gene remains relatively unchanged for *GCN5* deleted cells relative to wild-type cells (Figure 3C). To determine if *DBP2* exon 2 length influences co-transcriptional Lea1 recruitment, we tested the recruitment of Lea1 to *DBP2* lacking the extension on exon 2. We find that the GFP extension has only a mild effect on the overall signal strength observed in the presence of *GCN5* with the primer sets used here, and recruitment of Lea1 is eliminated when *GCN5* is deleted regardless of whether exon 2 is extended (data not shown).

Our discovery of an essential requirement for Gcn5's HAT activity in its interaction with Lea1/Msl1 led to the prediction that its HAT activity would also be required for the co-transcriptional recruitment of Lea1, and this is what is observed. When Gcn5's HAT activity is abrogated by the KQL mutation, no co-transcriptional recruitment of Lea1 is observed, whereas Lea1's association is unaffected by the LKN mutation (Figure 3D). Pol II occupancy is not significantly affected by either mutation (Figure 3E).

A somewhat trivial explanation of these results is that *GCN5* deletion or elimination of its HAT activity decreases the amount of Lea1, leading to a decrease in its association with the gene. However, total Lea1 protein levels are unchanged in the absence of Gcn5. Neither are levels of Msl1 protein altered (Figure 3F).

**Figure 4:**
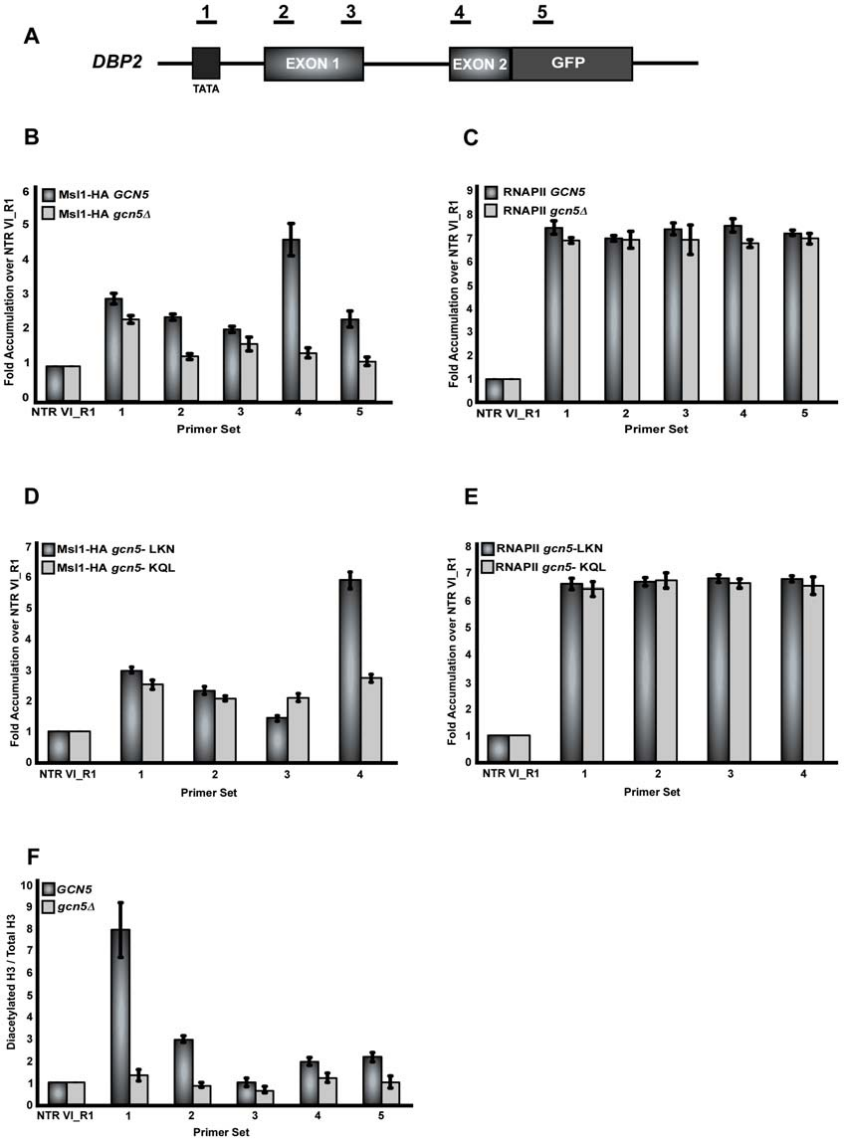
Deletion of *GCN5* affects co-transcriptional recruitment of Msl1 to *DBP2*. (A) Schematic of the intron-containing gene, *DBP2*. Underlined numbers represent the amplicons generated from each primer set used in the study. (B) Graph depicting Msl1 occupancy within each region of *DBP2* relative to the non-transcribed region, with wild-type or *gcn5Δ*. Dark grey bars represent Msl1 occupancy in the presence of wild-type *GCN5* and light grey bars represent Msl1 occupancy in the *gcn5Δ* strain. (C) Bar graph depicting RNAP II occupancy within *DBP2*. Dark grey bars represent RNA pol II occupancy in the *MSL1-HA* strain and light grey bars represent RNAP II occupancy in the *MSL1-HA gcn5*Δ. (D) Graph depicting the Msl1 occupancy in the presence of the Gcn5 HAT mutants, LKN and KQL. Dark grey bars represent the levels of Msl1 with the Gcn5 LKN mutation, light grey bars represent Msl1 with the Gcn5 mutant KQL. (E) Bar graph depicting the occupancy of RNA pol II within *DBP2* in the presence of the Gcn5 HAT mutants. Dark grey bars represent RNAP II with the Gcn5 LKN mutation and light grey bars represent RNAP II with the Gcn5 KQL mutant. (F) ChIP analysis of histone H3 K9/14 acetylation within *DBP2* in wild-type and *gcn5Δ* strains using an antibody directed against diacetylated histone H3 (Upstate). Dark grey bars represent wild-type and light grey bars represent histone acetylation in a *gcn5Δ* strain. Data are represented as diacetylated histone H3 normalized to the total amount of histone H3 (Total H3). All graphs depict the average of at least three independent experiments, and error bars represent the standard deviation.

Co-transcriptional recruitment of Msl1 to *DBP2* was also examined. As previously described, Msl1 association with *DBP2* is also enriched in regions downstream of the branchpoint sequence. This enrichment is abrogated when *GCN5* is deleted or when Gcn5 HAT activity is eliminated (Figure 4B and 4D, respectively). Consistent with previous studies, we routinely observe that the fold enrichment of Msl1 near the branchpoint (primer set 4) relative to the nontranscribed control is lower than for Lea1. Again RNA polymerase II occupancy was not significantly altered in the strain deleted of *GCN5* (Figure 4C). To examine the specificity of the enrichment of Msl1 within *DBP2*, we examined the recruitment of Msl1 (and Lea1) to a region further upstream of the promoter of *DBP2* and find that neither protein is significantly recruited to these regions in the presence or absence of *GCN5* (Figure S1B), suggesting that recruitment of Lea1 and Msl1 is transcription dependent. These data demonstrate that co-transcriptional Msl1 and Lea1 recruitment to the branchpoint region of the pre-mRNA is dependent upon *GCN5*.

To determine if Gcn5 affects splicing of *DBP2*, we performed qRT-PCR to determine the ratio of unspliced pre-mRNA to total *DBP2* RNA. Using this analysis, we reproducibly detect an approximately two-fold increase in the Precursor/Total RNA ratio in *GCN5* deleted cells compared to WT cells (Figure S2A). When the genes encoding the splicing factors Msl1 and Lea1 are deleted, we observe a 10–15 fold increase in Precursor/Total RNA ratio relative to WT (approximately 5–9% total unspliced) (Figure S2B). While deletion of *GCN5* leads to a moderate increase in intron accumulation when compared to deletion of a bona fide splicing factor, this reproducible increase indicates that splicing of *DBP2* is sensitive to the absence of Gcn5. While it is clear that post-transcriptional splicing can occur [8], at least under optimal growth conditions, when co-transcriptional splicing is abrogated, it is likely that the additive effect of disrupting co-transcriptional splicing across the genome has important implications for optimal cellular function, particularly under conditions in which optimal splicing of particular genes is required for cell viability. This hypothesis is currently being tested.

Interestingly, we consistently observe enrichment of Msl1 upstream of exon 1, within the promoter region of *DBP2*, which is illustrated by the amplification observed with primer set 1 (Figure 4B, compare to primer set 4, which depicts peak enrichment within the gene). The level of Msl1 in this region is only mildly decreased when *GCN5* is deleted or its catalytic activity is eliminated. This result is surprising since it suggests that the protein is associated with the chromatin before synthesis of the appropriate RNA signal and that the crosslinking step has captured branchpoint-independent interactions between Msl1 and the transcription complex.

Msl1, but not Lea1, has been shown by yeast two-hybrid to interact with Ssl2, a component of TFIIH, and Tra1, a SAGA subunit that interacts with acidic activators [35]. Furthermore, Msl1, but not Lea1, affinity purifies with TAF4, a subunit of the TFIID complex [36]. These unique interactions between Msl1 and components of the transcription machinery that are predicted to act at or near the promoter suggest that Msl1 may be recruited early during transcription initiation and could form a bridge between transcription and U2 snRNP recruitment.

### Gcn5 affects acetylation of *DBP2-*bound histones

The finding that Gcn5 HAT activity is required for co-transcriptional recruitment of the U2 snRNP to *DBP2* leads to the prediction that acetylation of *DBP2-*bound histones is also Gcn5 dependent. To test this prediction, ChIP was performed using an antibody that recognizes diacetylated histone H3. Histone H3 acetylation peaks at the promoter region of *DBP2* (Figure 4F) with little evidence of enriched acetylation in the body of the gene. This acetylation drops dramatically when *GCN5* is deleted, demonstrating that *DBP2*-bound histones are acetylated in a Gcn5-dependent manner. Since histone deacetylases (HDACs) have been shown to affect rapid/dynamic histone acetylation patterns we examined histone acetylation in the absence of the HDACs shown in Figure 2C, namely Rpd3, Hos2, and Hos3. We found that deletion of these HDACs did not significantly affect acetylation at the promoter or in the body of the gene (data not shown). It remains possible that other deacetylases or some combination of HDACs may contribute to regulation of histone marks involved in co-transcriptional splicing. It is also possible that histones are being rapidly exchanged such that the relevant marks within the body of the gene that facilitate co-transcriptional recruitment of Msl1 and Lea1 are difficult to detect. Nonetheless, Gcn5's acetylation activity, most likely toward histones, appears to be a critical determinant of Msl1 and Lea1 recruitment to the branchpoint. The precise role of Gcn5-mediated acetylation of lysine residues on either histone (H3, H2B, or H4) or non-histone substrates is currently under investigation.

### Co-transcriptional recruitment of the U1 snRNP, but not the U5 snRNP, occurs in the absence of Gcn5

**Figure 5:**
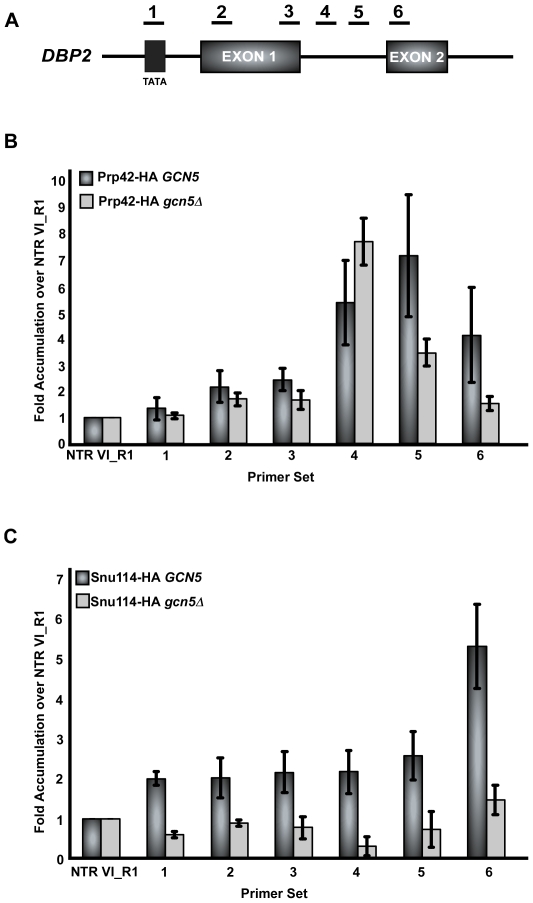
Co-transcriptional recruitment of U1 snRNP and U5 snRNP in the presence and absence of *GCN5*. (A) Schematic of the intron-containing gene, *DBP2*. Underlined numbers represent the amplicons generated from each primer set used in the study. (B) Bar graph depicting recruitment of U1 snRNP (Prp42-HA) in the presence and absence of *GCN5*. Dark grey bars represent the occupancy of Prp42-HA in the presence of wild-type *GCN5* and light grey bars represent Prp42-HA in the absence of *GCN5*. Occupancy is measured as fold accumulation over the non-transcribed region. (C) Bar graph depicting the recruitment of U5 snRNP (Snu114-HA) in the presence and absence of *GCN5*. Dark grey bars represent the Snu114-HA in the presence of *GCN5*, and light grey bars represent Snu114-HA occupancy in the absence of *GCN5*. Graphs represent the average of at least three independent experiments, and error bars represent the standard deviation.

Co-transcriptional recruitment of the spliceosome to the emerging pre-mRNA has been shown to occur in a stepwise fashion [8],[9]. Here we show that deletion of *GCN5* severely abrogates the co-transcriptional recruitment of the U2 snRNP. Combined with our genetic analysis, these results strongly suggest a specific role for Gcn5 activity in U2 snRNP function. Nonetheless, it is possible that deletion of *GCN5* acts generally to disrupt co-transcriptional recruitment of all snRNPs. To address this, recruitment of a representative component of the U1 snRNP and triple snRNP was examined. Chromatin IP of Prp42 has been shown to be an indicator of U1 snRNP recruitment to intron-containing genes [7],[8]. To determine if recruitment of the U1 snRNP is altered in the absence of *GCN5*, Prp42 association with *DBP2* was analyzed. The U1 snRNP associates with the *DBP2* pre-mRNA shortly after synthesis of the 5′ splice site, consistent with reports by others (Figure 5B) [7],[8]. Unlike its effect on U2 snRNP recruitment, deletion of *GCN5* does not abrogate the recruitment of the U1 snRNP, demonstrating that the U1 snRNP is still being actively recruited to the pre-mRNA in a co-transcriptional manner (Figure 5B). Hence, the observed disruption of co-transcriptional recruitment of the U2 snRNP in the absence of Gcn5's catalytic activity is specific, and *GCN5* deletion does not abrogate all early steps in spliceosome assembly.

Since co-transcriptional spliceosome assembly occurs in a stepwise fashion, the prediction is that disruption of U2 snRNP recruitment due to deletion of *GCN5* would affect co-transcriptional spliceosome assembly downstream of the U2 snRNP. Snu114 is a U5 snRNP protein that is involved in the destabilization of U1 and U4 snRNAs during spliceosome assembly [37]–[39]. Chromatin IP of Snu114 shows that the U5 snRNP is enriched downstream of the 3′ splice site, a result consistent with previous observations (Figure 5C) [8]. However, deletion of *GCN5* eliminates the co-transcriptional recruitment of U5 snRNP (Figure 5C), indicating that the lack of U2 snRNP recruitment does alter the recruitment of downstream factors and cripples spliceosome assembly. Although this is consistent with the ordered assembly model of co-transcriptional splicing [8],[9], we cannot rule out the possibility of an independent effect by Gcn5 on U5 recruitment.

### Gcn5 affects co-transcriptional recruitment of Lea1 and Msl1 to *ECM33* and acetylation of its promoter bound histones

**Figure 6:**
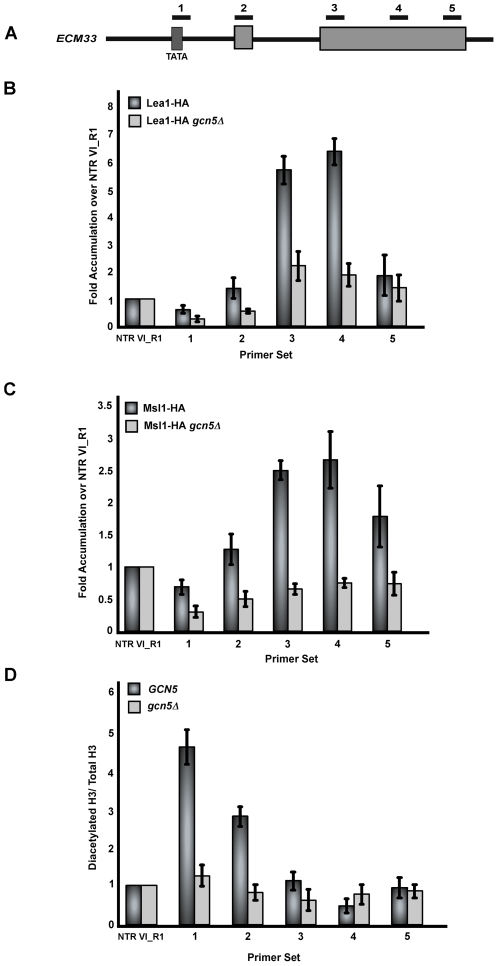
Deletion of *GCN5* affects co-transcriptional recruitment of Msl1 and Lea1 to *ECM33* and histone H3 acetylation. (A) Schematic of the intron-containing gene, *ECM33*. Underlined numbers represent the amplicons generated from each primer set used in the study. (B) Graph depicting the occupancy of Lea1 at each region of *ECM33* relative to the non-transcribed region, in wild-type or *gcn5Δ*cells. Dark grey bars represent Lea1 with wild-type *GCN5*, and light grey bars represent Lea1 levels in the *gcn5Δ* strain. (C) Bar graph depicting Msl1-HA occupancy within *ECM33* relative to the non-transcribed control. Dark grey bars represent Msl-HA with wild-type *GCN5* and light grey bars represent Msl1-HA occupancy in the *gcn5*Δ strain. Data are represented as fold accumulation over the non-transcribed region. (D) ChIP analysis of histone H3 K9/14 acetylation in *ECM33* of wild-type and *gcn5Δ* strains using an antibody against diacetylated histone H3. Dark grey bars represent wild-type and light grey bars represent histone acetylation in a *gcn5Δ* strain. Data are represented as diacetylated histone H3 normalized to the total amount of histone H3 (Total H3). Graphs depict the average of three independent experiments, and error bars represent the standard deviation.

*DBP2* was chosen for these studies because of its previously-characterized suitability for chromatin IP studies. *DBP2*'s long intron (∼1 Kb) and long first exon (∼1 Kb) allow for resolution of protein association throughout the gene. We wanted to examine a second well-characterized, intron-containing gene to determine if Gcn5's role in co-transcriptional recruitment of Lea1 and Msl1 is more general. *ECM33* has previously been described by others to be a gene to which splicing factors, including the U2 snRNP, are co-transcriptionally recruited [8]. Examination of the co-transcriptional recruitment of Msl1 and Lea1 to *ECM33* in the presence of Gcn5 revealed that Lea1 and Msl1 recruitment occurred after the formation of the branchpoint (Figure 6B and 6C, respectively), consistent with what we observed with *DBP2*. In the absence of *GCN5*, recruitment of Lea1 and Msl1 was abolished (Figure 6B and 6C, respectively). A third gene, *YRA1* shows a similar Gcn5-dependent pattern of Msl1 and Lea1 recruitment (data not shown). As with *DBP2*, when recruitment of Msl1 and Lea1 to a region further upstream of the promoter of *ECM33* was examined in the presence and absence of *GCN5*, *we* find that neither protein is significantly recruited to this region, reinforcing the transcription-dependence of their recruitment (Figure S1D). Also similar to *DBP2*, deletion of *GCN5* leads to an increase in the Precursor/Total RNA ratio when compared to WT cells (approximately 4–5 fold) (Figure S2A).

We next examined the acetylation pattern of *ECM33-*bound histones by ChIP. Consistent with what we observed with *DBP2*, a strong Gcn5-dependent peak in acetylation was observed at the promoter of *ECM33*, (Figure 6D) and little change in this pattern was observed when the HDACs were deleted (data not shown).

As with *DBP2*, Msl1 recruitment peaks after synthesis of the branchpoint of *ECM33* (Figure 6C, primer sets 3–4). Additionally, analysis of the upstream region of *ECM33* shows some early association of Msl1 relative to the non-transcribed control (Figure 6C) and especially relative to the peak in signal with primer sets 3 and 4. This early association of Msl1 is most evident using primer set 2, although the short distance between amplicons 1 and 2 (around 300 base pairs) is likely too small to completely resolve. Nonetheless, the early recruitment of Msl1 to *ECM33* is less pronounced than what we observe with *DBP2*. A possible explanation for this is the transcriptional frequency of the individual genes. For example, *DBP2* generates about 4 times the number of mRNA molecules as *ECM33*, and the transcriptional frequency is approximately 7 times greater [40]. It is possible that the increase in transcription of *DBP2* allows for more recruitment of Msl1 to the promoter.

Taken together, these results suggest that Gcn5-dependent co-transcriptional recruitment of Msl1 and Lea1 to the branchpoint is a common feature among intron-containing genes.

## Discussion

Recent work from a number of groups provides evidence of spatial and temporal coordination of transcription and pre-messenger RNA processing. Simultaneously, there has been an emerging understanding of the role of histone modification and the enzymes that catalyze these modifications in regulating gene expression. Here we demonstrate a new function for the histone acetyltransferase Gcn5. In addition to its previously-characterized role in transcriptional activation, Gcn5 can specifically affect co-transcriptional assembly of the spliceosome onto constitutively-spliced genes (Figure 3, Figure 4, Figure 5, Figure 6). Our genetic analysis reveals that Gcn5 has functional interactions with two specific U2 snRNP components Msl1 and Lea1, and these functional interactions depend on Gcn5's HAT activity (Figure 1 and Figure 2). Our genetic analysis further provides evidence of the specificity of this interaction and suggests that it most likely occurs within the context of a functional SAGA complex that is targeted to chromatin (Table 1). These studies demonstrate a novel mechanism whereby a protein complex whose catalytic activity establishes a mark of active transcription also plays a central role in co-transcriptional mRNA processing.

### How does Gcn5 affect spliceosome assembly?

In recent years, there has been strong evidence that splicing can occur co-transcriptionally in yeast and in mammals. However, the mechanism by which spliceosome assembly is coordinated with transcription has been difficult to decipher, particularly in yeast. The genetics and ChIP results described above suggest a model in which Gcn5 mediates co-transcriptional spliceosome assembly by affecting histone acetylation. While we have not detected U2 snRNP acetylation, these data do not rule out the possibility that an additional non-histone substrate (or substrates) of Gcn5 can affect co-transcriptional spliceosome assembly, which is something that we continue to explore. It would nonetheless be interesting if Gcn5's acetylation activity is targeted toward a non-histone substrate to abrogate co-transcriptional splicing.

Since previous studies have shown that Gcn5 can affect transcription elongation [16], it is possible that Gcn5 effects on transcription elongation could be responsible for its role in co-transcriptional splicing, especially in light of studies that indicate that changes in elongation can influence pre-mRNA splicing [11],[12],[41]. Nonetheless, several lines of evidence suggest that it is not a Gcn5 effect on elongation per se that underlies its role in co-transcriptional snRNP recruitment. First, *GCN5* deletion does not appear to significantly affect pol II levels throughout *DBP2 or ECM33*. Furthermore, the genetic interactions between *MSL1* or *LEA1* and *GCN5* are not observed with the histone acetyltransferase that acts during elongation, *ELP3*. In light of these findings we favor a model in which Gcn5-dependent histone acetylation at the promoter facilitates co-transcriptional recruitment of splicing factors to the branchpoint. We think that it is likely that high promoter acetylation facilitates loading of a factor or factors onto elongating RNA polymerases, and these factors then recruit Msl1 and/or Lea1 to the branchpoint.

We cannot rule out that direct interactions between Gcn5 and the U2 snRNP may be important for recruitment, particularly since Gcn5 has been shown to associate both at the promoter and within the body of genes. Although our initial studies do not detect a direct association between Gcn5 and either Msl1 or Lea1, the interactions may be too weak or transient to detect biochemically.

### The role of co-transcriptional splicing in mature message formation

The analysis reported here indicates that deletion of Gcn5 leads to a reproducible increase in unspliced RNA relative to WT cells for both of the genes analyzed. While the amount of unspliced message that accumulates in the absence of Gcn5 is modest on a per gene basis, it is likely that the additive effect across the genome of decreased splicing efficiency when co-transcriptional splicing is abrogated is important. A number of studies of splicing in yeast have found, as we do, that some post-transcriptional splicing can occur even when co-transcriptional splicing is eliminated. Co-transcriptional recognition of splice signals is thought to be a means of increasing the efficiency and perhaps the rate of splicing. Hence, it is likely that conditions under which optimal splicing is necessary will be particularly sensitive to changes in co-transcriptional splicing, which we are currently exploring. We are also testing whether this Gcn5-dependence for optimal splicing increases under growth conditions in which the cell's transcription is particularly dependent on SAGA, which is reported to be the case under a variety of stress conditions [42].

### Branchpoint recognition is a critical step in coordinating splicing with transcription

Proper splicing is achieved by sequential recognition of the branchpoint by numerous factors, including the branchpoint binding protein (BBP) and the U2 snRNA (with its associated collection of snRNP-specific proteins). The exchange of BBP for the U2 snRNA is the first ATP-dependent step in splicing, and splice sites are committed to participate in this first ATP-dependent step when spliceosomal rearrangements lock the U2 snRNA into place [43]. The work described here suggests that branchpoint recognition is a critical step in coordinating splicing with transcription. A recent mammalian study also suggests that branchpoint recognition is closely tied to transcription. This study identified interactions between U2 snRNP components and the H3K4me3 interacting protein Chd1. Chd1 bridges U2 snRNP association with trimethylated histone H3, indicating that U2 snRNP recruitment in mammals is closely tied with transcription and specifically with chromatin “marks” of active transcription [44].

Evidence that a transcriptional coactivator that functions at the 5′ end of the gene can influence U2 snRNP recruitment is particularly interesting in light of a recent proposal that the majority of second exons in yeast may be too short to support stable recruitment of the U2 snRNP and, as a consequence, most endogenous yeast gene splicing is completed post-transcriptionally. Our results suggest that the activity of Gcn5 facilitates co-transcriptional recruitment of the U2 snRNP to at least a subset of genes. Furthermore, co-transcriptional U2 snRNP recruitment may even involve recruitment of Msl1 before synthesis of the branchpoint since Msl1 appears to have unique interactions with the transcription machinery. Our data suggest that the commitment to splicing is likely made co-transcriptionally, and Gcn5 facilitates U2 snRNP association with the pre-mRNA to allow a fluid transition to a U2 snRNP poised to participate in post-transcriptional splicing catalysis.

Studies of the mammalian counterpart of SAGA suggest that interactions between the complex and the U2 snRNP may be evolutionarily conserved. Martinez *et al*. reported that a U2 snRNP protein copurified with the human STAGA complex, although the functional significance of this interaction was not clear [19]. Our results help to explain the functional link between the chromatin modifying machinery and pre-mRNA splicing and demonstrate that Gcn5, likely within the context of the SAGA complex, has a previously undescribed activity in pre-mRNA splicing.

## Materials and Methods

### Yeast strains, media, and DNA constructs

All *S. cerevisiae* strains used in this study are listed in Table S1. Strains described in Table S1 are in the BY4743 strain background, with the exception of Lea1-HA and Msl1-HA strains used for ChIP assays, provided by Karla Neugebauer. All strains were propagated according to standard procedures in either rich media (YPD) or appropriate selective media. Plasmid shuffling was performed on 5- fluoroorotic acid (5-FOA) plates. Standard methods for mating, sporulation, transformations, and tetrad analysis were used as described in *Methods in Yeast Genetics: A Cold Spring Harbor Laboratory Course Manual*. The genotype of each viable spore was confirmed by PCR. Plasmids used in this study are listed in Table S2.

### Viability assay/dilution series

For growth analysis, strains containing a wild-type copy of *GCN5* on a centromeric pRS316 (*URA3*) plasmid were selected for plasmid loss on 5-FOA. Strains were then grown overnight in YPD media at 30°C. Cells were diluted to an O.D._600_ of 0.1 in 10 ml of YPD, and incubated at 30°C until all strains reached an O.D._600_ of 0.35. A ten-fold serial dilution of each strain was spotted onto YPD plates and incubated 3–5 days at 30°C.

### Yeast whole cell extract/western blot analysis

Cells were grown to an O.D._600_ of 1.0 and lysed using FA-1 Lysis buffer (50 mM HEPES-KOH pH 7.5, 140 mM NaCl, 1 mM EDTA pH 8.0, 1% Triton-X, 0.1% Deoxycholate, plus protease inhibitors) and 0.5 mm glass beads with 5 minutes of vortexing at 4°C. The supernatant was cleared by centrifugation and protein concentration was determined by Bradford Assay (Bio-Rad). 50 µg of total protein was fractionated by SDS-PAGE electrophoresis and transferred to a nitrocellulose membrane for immunoblotting with 1∶2000 dilution of anti-PGK1 (Molecular Probes) and 1∶1000 dilution of anti-HA 12CA5 (Roche), followed by chemiluminescent detection (Pierce).

### Chromatin immunoprecipitation (ChIP)

Cells were grown in YPD to an O.D._600_ 0.5–0.7 and then crosslinked for 15 minutes with formaldehyde to a final concentration of 1%. Cells were disrupted with glass beads (0.5 mm) for 40 minutes at 4°C and lysates were cleared by centrifugation. To shear chromatin, lysates were sonicated for a total of six minutes at 30% intensity (15 seconds on, 15 seconds off, and on ice). After sonication, samples were precleared with CL4B Sepharose beads (Sigma). The precleared samples were then used for immunoprecipitation with either 12CA5 (Roche) antibody against the HA epitope or 8WG16 (Covance) antibody against RNA pol II. After immunoprecipitation, samples were washed and incubated overnight at 65°C to reverse crosslinking, followed by incubation with Proteinase K (Sigma). DNA was purified using a PCR product purification kit (Qiagen) and analyzed by real-time PCR. Input DNA was diluted 1∶20 and 1 µl of this was used in a 25 µl reaction volume. For ChIP DNA, samples were diluted 1∶5 and 1 µl of this was used in a 25 µl reaction volume. Reactions consisted of 12.5 µl SYBR GREEN Master Mix (Applied Biosystems) and 0.5 µM Primers. Real time PCR was performed using an ABI7700 (Applied Biosystems). All samples were run in triplicate for each independent experiment.

For quantification, standard curves were generated for each primer set, and DNA concentration for each INPUT and ChIP sample was calculated. ChIP values were divided by the INPUT, and these values were divided by the non-transcribed control and expressed as fold accumulation over the non-transcribed control. Reported values are averages of at least three independent experiments, and error bars represent the standard deviation.

For ChIP experiments in Figure 4F and Figure 6D, the ChIP protocol described above was used except samples were sonicated for seven minutes at 30% intensity (15 seconds on, 15 seconds, off, and on ice). Samples were used for immunoprecipitation with either anti-acetylated histone H3 (Upstate 06-599) or anti-histone H3 (AbCam ab1791) overnight at 4°C.

For quantification, standard curves were generated for each primer set. DNA concentration for each INPUT and ChIP sample was calculated using these standard curves and normalized to the non-transcribed control VI_R1. The normalized IP values calculated for acetylated H3 were divided by the normalized IP values calculated for total H3. These values are expressed as diacetylated H3 over total Histone H3. Reported values are averages of three independent experiments, and error bars represent the standard deviation.

The data in Figure 5 was generated by standard PCR analysis, ethidium bromide staining, and quantification. The reaction volume was 50 µl, with 0.75 µl of template for INPUT, and 5 µl of template for ChIP DNA. Primers were used at a final concentration of 1 µM. PCR products were analyzed on a 1.75% agarose gel. Results were quantified using ImageQuant software (Molecular Dynamics). Primer sequences are listed in Table S3, S4, S5.

Materials and methods for Figure S1 and Figure S2 are provided in Text S1.

## Supporting Information

Figure S1 Recruitment of Msl1 and Lea1 to *DBP2* and *ECM33* is dependent on transcription. (A) Schematic of chromosome XIV and relative location of *DBP2*. Underlined numbers represent amplicons from each primer set used in this study. (B) Graph represents occupancy of Lea1 and Msl1 at each region of *DBP2* relative to the non-transcribed region in the presence and absence of *GCN5*. Dark grey bars represent Lea1/Msl1 recruitment in the presence of *GCN5* and light grey bars represent recruitment of Lea1/Msl1 in the absence of *GCN5*. (C) Schematic of chromosome II and the relative location of *ECM33*. Underlined numbers represent amplicons from each primer set used in this study. (D) Occupancy of Lea1 and Msl1 at *ECM33* relative to the non-transcribed region. Dark Grey bars represent Lea1/Msl1 recruitment in the presence of *GCN5* and light grey bars represent Lea1/Msl1 recruitment in its absence. Graphs depict the average of three independent experiments, and error bars represent the standard deviation.
(1.19 MB TIF)

Figure S2 Deletion of *GCN5* alters splicing of *DBP2* and *ECM33* transcripts. Quantitative RT-PCR of *DBP2* and *ECM33* in the absence of *GCN5*, *MSL1*, or *LEA1*. (A) Graph represents the ratio of precursor *DBP2* or *ECM33* transcript relative to mature message in wild type and *GCN5* deleted cells. Data is represented as a ratio of precursor (unspliced) RNA to total message. (B) Graph represents the ratio of precursor (unspliced) RNA to total *DBP2* or *ECM33* message in wild type, *MSL1* deleted, and *LEA1* deleted cells. Error bars represent the standard deviation.
(0.25 MB TIF)

Table S1 List of yeast strains used in this study.
(0.06 MB PDF)

Table S2 List of plasmids used in this study.
(0.03 MB DOC)

Table S3 *DBP2* primers used for ChIP (Figure 5).
(0.03 MB DOC)

Table S4 *DBP2* and *ECM33* primers used for ChIP real time PCR analysis.
(0.04 MB DOC)

Table S5 *DBP2* and *ECM33* primers used for quantitative RT-PCR (Figure S2).
(0.03 MB DOC)

Text S1 Supplemental materials and methods.
(0.04 MB DOC)
