# Supplementary figures and images for: Acetylation by the Transcriptional Coactivator Gcn5 Plays a Novel Role in Co-Transcriptional Spliceosome Assembly
Source: PLoS Genet. 2009 Oct 16;5(10):e1000682. doi: 10.1371/journal.pgen.1000682 (PMC2752994; doi:10.1371/journal.pgen.1000682)

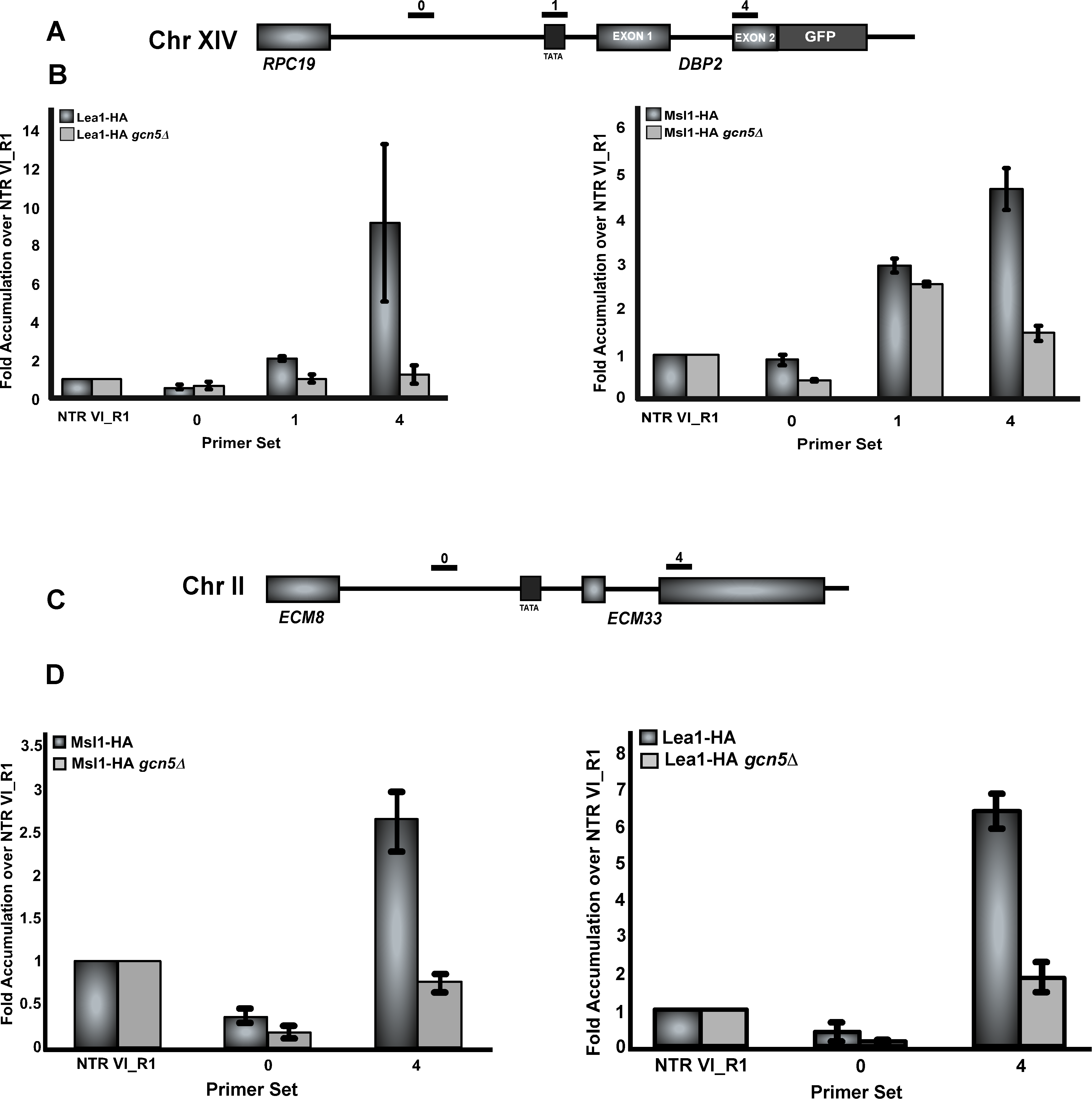

Supplement: Figure S1 — Recruitment of Msl1 and Lea1 to DBP2 and ECM33 is dependent on transcription. (A) Schematic of chromosome XIV and relative location of DBP2. Underlined numbers represent amplicons from each primer set used in this study. (B) Graph represents occupancy of Lea1 and Msl1 at each region of DBP2 relative to the non-transcribed region in the presence and absence of GCN5. Dark grey bars represent Lea1/Msl1 recruitment in the presence of GCN5 and light grey bars represent recruitment of Lea1/Msl1 in the absence of GCN5. (C) Schematic of chromosome II and the relative location of ECM33. Underlined numbers represent amplicons from each primer set used in this study. (D) Occupancy of Lea1 and Msl1 at ECM33 relative to the non-transcribed region. Dark Grey bars represent Lea1/Msl1 recruitment in the presence of GCN5 and light grey bars represent Lea1/Msl1 recruitment in its absence. Graphs depict the average of three independent experiments, and error bars represent the standard deviation. (1.19 MB TIF) [file pgen.1000682.s001.tif]

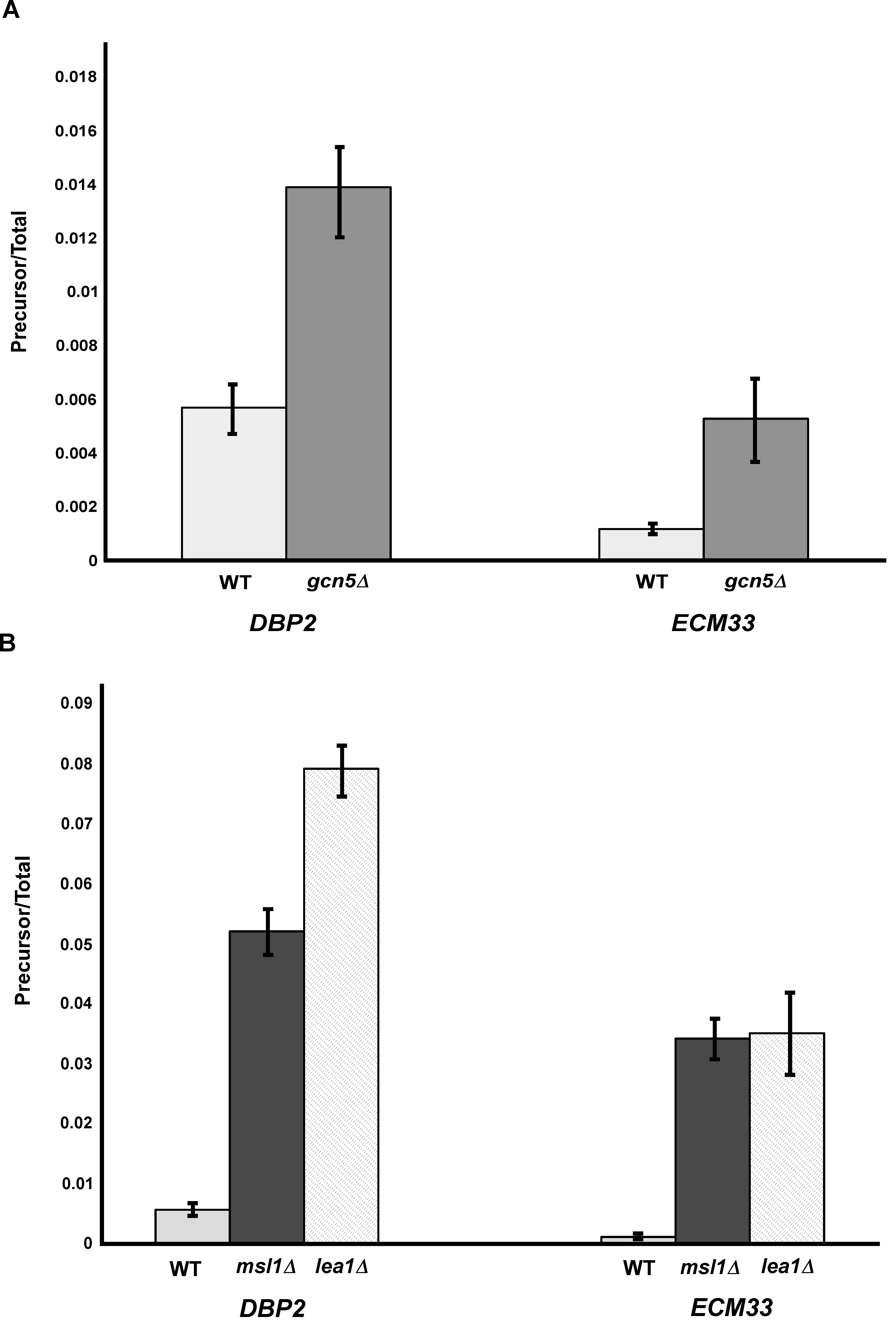

Supplement: Figure S2 — Deletion of GCN5 alters splicing of DBP2 and ECM33 transcripts. Quantitative RT-PCR of DBP2 and ECM33 in the absence of GCN5, MSL1, or LEA1. (A) Graph represents the ratio of precursor DBP2 or ECM33 transcript relative to mature message in wild type and GCN5 deleted cells. Data is represented as a ratio of precursor (unspliced) RNA to total message. (B) Graph represents the ratio of precursor (unspliced) RNA to total DBP2 or ECM33 message in wild type, MSL1 deleted, and LEA1 deleted cells. Error bars represent the standard deviation. (0.25 MB TIF) [file pgen.1000682.s002.tif]
