# Supplementary material for: Acetylation by the Transcriptional Coactivator Gcn5 Plays a Novel Role in Co-Transcriptional Spliceosome Assembly
Source: PLoS Genet. 2009 Oct 16;5(10):e1000682. doi: 10.1371/journal.pgen.1000682 (PMC2752994; doi:10.1371/journal.pgen.1000682)
Supplement: Table S1 — List of yeast strains used in this study. (0.06 MB PDF) [file pgen.1000682.s003.pdf]

Table S1. List of yeast strains used in this study

Name	Parent	Relevant Phenotype	Reference
TJY1701	BY4743	*MATα his3Δ1 leu2Δ0 lys2Δ0 ura3Δ0*	Open Biosystems
TJY0386	BY4741	*gcn5Δ::KanMX4*	Open Biosystems
TJY0396	BY4742	*msl1Δ::KanMX4*	Open Biosystems
TJY0462	BY4742	*lea1Δ::KanMX4*	Open Biosystems
TJY0408	BY4742	*cus1Δ::KanMX4 [URA3 CUS1]*	Open Biosystems
TJY0547	BY4742	*cus1Δ::KanMX4 [LEU2 CUS 1-54]*	Open Biosystems
TJY2042	BY4741	*spt3Δ::KanMX4*	Open Biosystems
TJY2043	BY4741	*spt7Δ::KanMX4*	Open Biosystems
TJY2044	BY4741	*spt8Δ::KanMX4*	Open Biosystems
TJY0499	BY4741	*sas3Δ::KanMX4*	Open Biosystems
TJY0500	BY4741	*ubp8Δ::KanMX4*	Open Biosystems
TJY0504	BY4741	*ada2Δ::KanMX4*	Open Biosystems
TJY2045	BY4741	*ada3Δ::KanMX4*	Open Biosystems
TJY0443	BY4741	*elp3Δ::KanMX4*	Open Biosystems
TJY0001	LG1	*LEA1-HA_3::TRP1 DBP2::GFP::KanMX4*	(Görnemann et al., 2005)
TJY0476	LG1	MSL1-*HA_3::TRP1*	(Görnemann et al., 2005)
TJY0447	BY4743	*gcn5Δ::KanMX4 msl1Δ::KanMX4*	This study
TJY0469	BY4743	*gcn5Δ::KanMX4 lea1Δ::KanMX4*	This Study
TJY0441	BY4743	*gcn5Δ::KanMX4 mud2Δ::KanMX4*	This study
TJY0457	BY4743	*gcn5Δ::KanMX4 cus2Δ::KanMX4*	This Study
TJY0543	BY4743	*gcn5Δ::KanMX4 cus1Δ::KanMX4 [LEU2 CUS1-54]*	This study
TJY0528	BY4743	*ada2Δ::KanMX4 msl1Δ::KanMX4*	This study
TJY0527	BY4743	*ada2Δ::KanMX4 lea1Δ::KanMX4*	This study
TJY0540	BY4743	*ada3Δ::KanMX4 msl1Δ::KanMX4*	This study
TJY0541	BY4743	*ada3Δ::KanMX4 leal1Δ::KanMX4*	This study
TJY0531	BY4743	*spt3Δ::KanMX4 msl1Δ::KanMX4*	This study
TJY0532	BY4743	*spt3Δ::KanMX4 lea1Δ::KanMX4*	This study
TJY0544	BY4743	*spt8Δ::KanMX4 msl1Δ::KanMX4*	This study

TJY0533	BY4743	*spt8Δ::KanMX4 lea1Δ::KanMX4*	This study
TJY0534	BY4743	*spt7Δ::KanMX4 msl1Δ::KanMX4*	This study
TJY0535	BY4743	*spt7Δ::KanMX4 lea1Δ::KanMX4*	This study
TJY0529	BY4743	*sas3Δ::KanMX4 msl1Δ::KanMX4*	This study
TJY0530	BY4743	*sas3Δ::KanMX4 lea1Δ::KanMX4*	This study
TJY0423	BY4743	*elp3Δ::KanMX4 msl1Δ::KanMX4*	This study
TJY0468	BY4743	*elp3Δ::KanMX4 lea1Δ::KanMX4*	This study
TJY0548	LG1	*LEA1-HA_3::TRP1 DBP2::GFP::KanMX4 gcn5Δ::KanMX4*	This Study
TJY0549	LG1	MSL1-*HA_3::TRP1 gcn5Δ::KanMX4*	This study
TJY0566	LG1	*PRP42-HA_3::HIS3*	(Görnemann et al., 2005)
TJY0567	LG1	*PRP42-HA_3::HIS3 gcn5Δ::KanMX4*	This study
TJY0575	LG1	*SNU114-HA_3::TRP1 DBP2::GFP::KanMX4 gcn5Δ::KanMX4*	This study
TJY1706	BY4742	*mud2Δ::KanMX4*	Open Biosystems
TJY0124	BY4742	*cus2Δ::KanMX4*	Open Biosystems
TJY2856	BY4743	*ubp8Δ::KanMX4 msl1D::KanMX4*	This study
TJY2852	BY4743	*ubp8Δ::KanMX4 lea1D::KanMX4*	This study
TJY0475	LG1	*SNU114-HA_3::TRP1 DBP2::GFP::KanMX4*	(Görnemann et al., 2005)
TJY2365	BY4741	*sgf11Δ::KanMX4 lea1Δ::KanMX4*	This study
TJY2366	BY4741	*sgf11Δ::KanMX4 lea1Δ::KanMX4*	This study
TJY2669	LG1	*MSL1-HA_3::TRP1 DBP2::GFP::KanMX4*	This study
TJY2685	LG1	*MSL1-HA_3::TRP1 DBP2::GFP::KanMX4 gcn5Δ::KanMX4*	This study
TJY0562	BY4743	*rpd3Δ::KanMx4 lea1Δ::KanMx4*	This study
TJY0563	BY4743	*rpd3Δ::KanMx4 msl1Δ::KanMx4*	This study
TJY3225	BY4743	*hos2Δ::KanMx4 msl1Δ::KanMx4*	This study
TJY3227	BY4743	*hos2Δ::KanMx4 lea1Δ::KanMx4*	This study
TJY3220	BY4743	*hos3Δ::KanMx4 msl1Δ::KanMx4*	This study
TJY3222	BY4743	*hos3Δ::KanMx4 lea1Δ::KanMx4*	This study
TJY0558	BY4743	*rpd3Δ::KanMx4*	This study
TJY3141	BY4743	*hos2Δ::KanMx4*	This study
TJY3144	BY4743	*hos3Δ::KanMx4*	This study
