# Supplementary material for: Acetylation by the Transcriptional Coactivator Gcn5 Plays a Novel Role in Co-Transcriptional Spliceosome Assembly
Source: PLoS Genet. 2009 Oct 16;5(10):e1000682. doi: 10.1371/journal.pgen.1000682 (PMC2752994; doi:10.1371/journal.pgen.1000682)
Supplement: Table S2 — List of plasmids used in this study. (0.03 MB DOC) [file pgen.1000682.s004.doc]

Table S2. List of plasmids used in this study

| **Plasmid Number** | **Plasmid Description** | **Plasmid backbone** | **Reference** |
| --- | --- | --- | --- |
| pLP1641 | GCN5 | pRS 316 | L. Pillus |
| pLP1520 | GCN5 KQL/AAA | pRS 414 | (Wang et al., 1998) |
| pLP1521 | GCN5 LKN/AAA | pRS 414 | (Wang et al., 1998) |
| pFG001 | GCN5 | pRS 414 | This study |
| pLP1523 | *GCN5* | pRS 315 | L. Pillus |
| pFG003 | GCN5 KQL/AAA | pRS 316 | This study |
| pFG004 | GCN5 LKN/AAA | pRS 316 | This study |
|  | CUS1 | pRS 316 | (Wells et al., 1996) |
|  | CUS1-54 | pRS 315 | (Wells et al., 1996) |
