# Supplementary material for: Acetylation by the Transcriptional Coactivator Gcn5 Plays a Novel Role in Co-Transcriptional Spliceosome Assembly
Source: PLoS Genet. 2009 Oct 16;5(10):e1000682. doi: 10.1371/journal.pgen.1000682 (PMC2752994; doi:10.1371/journal.pgen.1000682)
Supplement: Table S3 — DBP2 primers used for ChIP (Figure 5). (0.03 MB DOC) [file pgen.1000682.s005.doc]

Table S3. *DBP2* primers used for ChIP (Figure 5)

| **Primer Name** | **Sequence** |
| --- | --- |
| DBP2 –379 F | 5’-CAC GCT AGT ATA GAT ACA GC-3’ |
| DBP2 –91 R | 5’-TAT TTG AGC GTA GGA CAG TC-3’ |
| DBP2 104 F | 5’-ACA GAC CAC AAG GCG GTA AC-3’ |
| DBP2 346 R | 5’-CGA AAG TGG TGA TTG GCT TT-3’ |
| DBP2 900 F | 5’-TTG ATG TGG TCT GCC ACT TG-3’ |
| DBP2 1078 R | 5’-CGT TGT CTT GAG AGG CTG TTT C-3’ |
| DBP2 1409 F | 5’-TGA CAA CCA TGA TAG TAC AGA AGA GAG-3’ |
| DBP2 1558 R | 5’-TTT CCG ATA CTC CCC ATC G-3’ |
| DBP2 1877 F | 5’-ATG CCG TCA TCC TTC TTG AC-3’ |
| DBP2 1970 R | 5’-TCG AAC TTG GGA TGC AAC AG-3’ |
| DBP2 2392 F | 5’-TTC ACC GAA CAA AAC AAA GG-3’ |
| DBP2 2612 R | 5’-CCA CCA TCT CTC TGC CTG TT- 3’ |
| NTR VI_R F | 5’-CAG GCA GTC CTT TCT ATT TC-3’ |
| NTR VI_R R | 5’-GCT TGT TAA CTC TCC GAC AG-3’ |
