# Supplementary material for: Acetylation by the Transcriptional Coactivator Gcn5 Plays a Novel Role in Co-Transcriptional Spliceosome Assembly
Source: PLoS Genet. 2009 Oct 16;5(10):e1000682. doi: 10.1371/journal.pgen.1000682 (PMC2752994; doi:10.1371/journal.pgen.1000682)
Supplement: Table S4 — DBP2 and ECM33 primers used for ChIP real time PCR analysis. (0.04 MB DOC) [file pgen.1000682.s006.doc]

# Table S4. *DBP2* and *ECM33* primers used for ChIP real time PCR analysis

| **Primer Name** | Sequence |
| --- | --- |
| DBP2 –379RT F | GGCGTATTCCGTATTGAATGAT |
| DBP2 –91RT R | GTATAAGTTATTTGAGCGTAGGACAGTC |
| DBP2 104RT F | TCGGTGGTCGTTCCAATTAC |
| DBP2 346RT R | ATCTCACTGTCCGATCTGTCG |
| DBP2 900RT F | TTGATGTGGTCTGCCACTTG |
| DBP2 1078RT R | CGTTGTCTTGAGAGGCTGTTTC |
| DBP2 2392RT F | GTGGCTATGGCCGTAGAGG |
| DBP2 2612RT R | TAGTTTGAACGACCTCTGTTACCC |
| GFP 104RT F | CTGTCAGTGGAGAGGGTGAAG |
| GFP 346RT R | CTGGGTATCTTGAAAAGCATTG |
| ECM33 -296F | aattttcggtagcgtgcttg |
| ECM33 -213R | tgcaagagaggtccgttgat |
| ECM33 +8F | attcaagaacgctttgactgct |
| ECM33 +127R | tcgagatttgtgaggaaagagg |
| ECM33 +454F | tccgctgctttggctagtat |
| ECM33 +560R | caccggtgattttcttgatagag |
| ECM33 +1075F | tggtggtgccattgaagttac |
| ECM33 + 1163R | gagtcgaagttagcaccacctc |
| ECM33 +1296F | GCTGCTGTTGGCGTTGCTTACTAT |
| ECM33 +1421R | AGTGATGAACCAACCGTCTCA |
| NTR VI_RRT F | CTAGTTGCACTAGGCGCAAAA |
| NTR VI_ RRT R | ACGCTTGCACTTGAAAAAGC |
| DBP2 -723F | TCTCACACAGGCAACATCTGA |
| DBP2-637E | TAACGAAGGGCATTTCCAAG |
| ECM33 -479F | ggagtgcaaaaacgggaaag |
| ECM33 -376R | taacgcggtttcagtcctgt |
