# Supplementary material for: Acetylation by the Transcriptional Coactivator Gcn5 Plays a Novel Role in Co-Transcriptional Spliceosome Assembly
Source: PLoS Genet. 2009 Oct 16;5(10):e1000682. doi: 10.1371/journal.pgen.1000682 (PMC2752994; doi:10.1371/journal.pgen.1000682)
Supplement: Table S5 — DBP2 and ECM33 primers used for quantitative RT-PCR (Figure S2). (0.03 MB DOC) [file pgen.1000682.s007.doc]

Table S5. *DBP2* and *ECM33* primers used for quantitative RT-PCR (Figure S2)

| **Primer Name** | **Sequence** |
| --- | --- |
| DBP2 EI_2.2F | TGAGACAATGTTAGTCCA |
| DBP2 EI_2.2R | AGTACCAGTACCTGCTCTACC |
| DBP2 Exon2_2F | CGTTATCAACTACGATATGCCAGG |
| ECM33 I260F | TCTCGTTGAGATGGTTTTGG |
| ECM33 E2_515R | CACCGGTGATGGTCAAGTTAC |
| ECM33 E2_435F | CTGCCACTGCTACTGCTCAA |
