# Supplementary material for: Acetylation by the Transcriptional Coactivator Gcn5 Plays a Novel Role in Co-Transcriptional Spliceosome Assembly
Source: PLoS Genet. 2009 Oct 16;5(10):e1000682. doi: 10.1371/journal.pgen.1000682 (PMC2752994; doi:10.1371/journal.pgen.1000682)
Supplement: Text S1 — Supplemental materials and methods. (0.04 MB DOC) [file pgen.1000682.s008.doc]

**Supplemental Material and Methods**

***Quantitative PCR***

Total cellular RNA was extracted by hot phenol-chloroform extraction. Prior to cDNA synthesis, total RNA was treated with DNase I (Promega) according to the manufacturer’s protocol. cDNA was synthesized from 1 μg of DNase-treated RNA in a 20 μl reaction mixture containing 1X First Strand Buffer, 2 mM each dNTP, 10 mM DTT, 2U RNasin (Promega), 1 μM gene-specific primer, and 200U of SuperScript II (Invitrogen). Quantitative PCR was performed using an ABI Prism 7700 Sequence Detector. Primer sequences are listed in Table S5. cDNA was diluted 1:20 and 1 μl of this was used in a 25 μl reaction volume. Reactions consist of 12.5 μl SYBR GREEN Master Mix (Applied Biosystems) and 0.5 μM Primers. All samples were run in triplicate for each independent experiment. Primers were designed to amplify unspliced (precursor) message using an intron-specific primer and total message amplification using primers specific to exon 2. Primer sequences are listed in Table S5.

## For quantification, standard curves were generated for each primer set, and to calculate ratio of precursor to total RNA, amount of unspliced (precursor) transcript was divided by the total amount of transcript (both spliced and unspliced).

## Chromatin Immunoprecipitation (ChIP)

Please refer to material and methods in main text.
